# Do the expressions about customarily doing reflect our cognition and emotion: evidence from Chinese BCC corpus

**DOI:** 10.3389/fpsyg.2025.1545253

**Published:** 2025-09-12

**Authors:** Limin Zhang, Zhaojie Lv, Longbo Ren

**Affiliations:** ^1^School of International Studies, Luoyang Institute of Science and Technology, Luoyang, China; ^2^School of Foreign Languages, Henan University of Science and Technology, Luoyang, China

**Keywords:** customarily, unbounded, bounded, cognitive processing, emotion

## Abstract

Through an in-depth analysis of the Chinese BCC colloquial corpus, this paper discusses the cognitive and emotional features of the temporal contouring of “customarily X” (X refers to verb or adjective) in spoken Chinese, focusing on the two different expressions of “often V” (V= verb) and “be often A” (A= adjective). Data analysis indicates that the “often V” structure is primarily used to express the frequent occurrence of actions, and its collocation with bounded verbs not only signifies the repetition of actions but also indexes the repetition of time on the timeline. The “often + unbounded verbs” changes the state of unbounded verbs and activates bounded cognitive processing. The “be often A” structure typically describes the regularity of emotions and physical sensations, and its frequent co-occurrence with negative adjectives serves to manifest the mental time of human cognition. These findings enrich the empirical basis for the study of temporal contouring in spoken Chinese and provide a nuanced analytical perspective for investigating the interface between cognitive mechanisms and affective dimensions in colloquial discourse.

## 1 Introduction

Language, cognition, and emotion have long been central themes in linguistic research. Understanding their interconnections is crucial for researchers aiming to explore how linguistic structures reflect cognitive processes and emotional states. Both emotion and language viewed by cognitive linguistics as intricate mental systems made up of several knowledge subsystems that interact in a variety of ways (Schwarz-Friesel, 2015). Cognitive linguistics emerged in the 1970s with the goal of integrating language and cognition to better understand how meanings are formed. Unlike Chomsky’s theory, which proposed a specialized mental module for language, cognitive linguistics contends that language knowledge is not separate from other cognitive processes. Instead, it is rooted in conceptual mechanisms and is shaped by our physical and environmental context.

Human language and cognition are tightly interwoven, with the notion that both have evolved simultaneously and are mutually dependent (Perlovsky, 2009). Lakoff (1988) highlighted that abstract concepts used by the mind to interpret the world possess a metaphorical structure. He argued that metaphors are not merely artistic devices, but rather cognitive mechanisms that the mind employs to generate new abstract meanings. Talmy (2000a) proposed the distinction between open and closed classes of linguistic forms. Open-class words, such as nouns and verbs, can be easily added to a language, often through borrowing from other languages. In contrast, closed-class forms, which include grammatical structures, remain stable over generations and are not easily borrowed. This distinction underscores the significant relationship between language and cognition. Closed-class forms are linked to cognitive concepts that have evolved over time through culture and language. As a result, many cognitive concepts are influenced by the linguistic constraints within which individuals and generations operate. Talmy suggested that closed-class forms play a more fundamental role in shaping cognition than words themselves, subtly affecting entire cultures. Language can have a profound impact on shaping or reshaping a fundamental domain such as spatial cognition (Majid et al., 2004).

On the other hand, affect and emotion are fundamental components of human experience. These states or feelings serve as indicators of personally significant events or circumstances, coloring our memories and thoughts (Rohr and Wentura, 2022). Emotion-related language input can highlight commonalities across diverse emotional events, thereby supporting the learning process (Brown, 1958; Hoemann et al., 2019). For instance, while expressions of sadness may vary, a child who hears the term “sad” applied to different situations might become more attuned to the underlying similarities. In this way, language is believed to facilitate emotional learning by helping children make sense of emotional variability across different contexts (Barrett, 2017). Language, especially the use of language related to emotions and mental states, plays a crucial role in social and emotional learning (Bell et al., 2024).

Therefore, language is not just a tool for communication; it also helps people connect emotions to specific situations, words, and experiences. Stern (1999) introduced “vitality contours” to describe the way emotions and feelings unfold over time, particularly in early infancy. In his work, Stern suggests that these temporal contours—essentially, the dynamic flow and rhythm of emotional experiences—serve as a fundamental building block for an infant’s social experience. In the light of this background, the objectives of this study are to investigate the cognitive and emotional features of customarily doing in Chinese spoken language. Specifically, the study aims to address the following research questions:

RQ1: What are the cognitive changes of verbs in the collocations of “often V”?

RQ2: What are the emotions behind the collocations of “be often A”?

To answer the research questions, we use Chinese BCC colloquial corpus as our materials and adopt Talmy’s state of boundedness as our principles when investigating the cognitive changes of verbs. Our study contributes to the literature in the following ways: first, it shows the boundedness and unboundedness in Chinese verbs. Second, the study discovers that the “be often A” is paired with more negative adjectives to reflect the mental time of human cognition. Taken together, these findings not only deepen our understanding of how lexical aspect interacts with frequency adverbs in Chinese, but also open new avenues for exploring the interface between language, cognition, and emotion in future corpus and experimental work.

## 2 Literature review

### 2.1 Habitual expressions and cognition

Language is not only the reflection of the world, but also shows what we think. Our habit can be conveyed through language. Hence, the customary expressions by people can reveal our cognition in the mind. The relationship between habit and cognition has been extensively studied in both psychology and linguistics. Habitual behaviors are often driven by cognitive automation processes, which are reflected in language. According to Duhigg (2012) in *The Power of Habit*, the formation of habits is determined by the reinforcement of neural circuits in the brain. Once a behavior becomes a habit, it becomes automated, reducing cognitive load. This cognitive automation can be manifested through habitual expressions in language, especially when describing daily behaviors. The use of frequent adverbs, such as “always” or “usually,” reflects the regularity of the behavior, making the language more concise and in line with habitual cognitive patterns.

Määttänen (2010) thought that habits are vehicles of cognition and habits of action are formed on the ground of past experience. Words are distinct units, formed by sequences of letters, which have the ability to represent or refer to something. Similarly, there exist internal components that convey mental content. Talmy (2000b, pp. 233-236) proposed 10 semantic types of temporal contouring event in his analysis of aspect mapping between Spanish and German, with the fifth type being “to customarily V.”

*Spanish*: soler V-INF

*German*: normalerweise V (present)/[früher/…] immer V(past)

Suele comer carne. / Solía comer carne.

Normalerweise isst er Fleisch. / Früher hat er immer Fleisch

gegessen.

“He eats meat.” / “He used to eat meat.” (Talmy, 2000b: 234)

Temporal contouring reflects the cyclical process of evolution in the real world (Ren, 2021), and language is a simulation of reality. Therefore, “to customarily V” is one of the core schemas of temporal contouring event. In English, the simple present tense is used to express actions or states that are habitual or regular, and also to indicate objective facts or universal truths. In Chinese, there are many frequency words to express habits, such as “经常” (often, customarily), “常常” (frequently), “时常”(regularly), and “总是” (always). The concept of time can be viewed as either linear motion or positional relationships in physics, with both involving conceptual metaphors. The structure of time is conceptualized as parallel to the structure of space, forming a conceptualization of event types through analogy with motion events. Therefore, the term “often” incorporates the concept of time, indicating the habitual occurrence of actions or the state of existence of something.

In corpus linguistics, researchers often reveal the cognitive characteristics of habitual expressions by analyzing large corpora. Frequently occurring action verbs in language (such as “do,” “make,” “have”) are closely associated with the expression of daily habits and can reflect the inherent cognitive patterns within a society and culture. Therefore, analyzing the habitual expressions based on the corpus can be vital to language research.

### 2.2 Language expressions and emotions

Language is a tool to explore emotions (Bamberg, 1997). Emotions to Wierzbicka (1995, p. 235) are a semantic domain, so we use nouns like *love, feel*, verbs like *to hate, fear* and adjectives like *happy, angry* to talk about emotions (Foolen, 2012). Harré and Gillett (1994) suggest that researchers study the way people use their emotion vocabulary in commenting on emotional displays and feelings. Harré and Gillett adhere to Stearns and Stearns’ (1985, 1988) idea of “emotionology” in their attempt to extract the underlying “theory of emotion” from the usage of the emotion lexicon (of a certain culture at a specific period). Emotions play a significant role in shaping language development and communication abilities. Understanding how individuals perceive and interpret emotions can provide insights into their language abilities and cognitive processes.

Researchers usually concentrate on descriptive emotion terms when considering the relationship between language and emotion (Majid, 2012). In Chinese, people particularly like to use adjectives to express their feeling and the state of things. Emotions are unique psychological states of humans, falling within the realm of responses in psychology (Zhao, 2008). The “*Cihai”* (a comprehensive Chinese encyclopedia) defines emotions as “feelings, referring to the psychological manifestations of human joy, anger, sorrow, and happiness. In a broad sense, emotions encompass feelings.” In Leech’s classification of seven types of linguistic meaning, affective meaning is one of them (Leech, 1981). Leech’s concept of affective meaning pertains to the feelings and attitudes of the speaker, indicating how language reflects the speaker’s personal emotions, including their attitudes towards the listener and the subject matter they are discussing. There are also interpretations of affective meaning within China. When people come into contact with and recognize objective things or phenomena, driven by utilitarian purposes, they inevitably incorporate their own subjective emotions into the cognitive outcomes. These subjective emotions then serve as an evaluative scale of affective judgment, which is integrated into semantics as an element, known as emotional meaning (Sun and Sun, 1998).

Vocabulary is the most crucial means of expressing emotions (Zhu, 1997). By using these emotional words, the emotions, attitudes, and inclinations of speakers and writers are conveyed. There are two ways in which vocabulary can contain emotional content:

(1) The vocabulary itself encapsulates emotions or moods, referring to various emotional states directed towards other people or things.
(2) The vocabulary expresses feelings, attitudes, and evaluations, or it points to positive or negative emotional aspects of things. Based on the nature of the emotion, words are generally categorized into positive terms (euphemisms), neutral terms, and negative terms (derogatory terms). Positive terms indicate that the speaker approves of the thing the word refers to. Negative terms suggest that the speaker disapproves of the thing the word denotes. Neutral terms, on the other hand, do not particularly convey the speaker’s emotions (Zhao, 2008).

### 2.3 Verb classification and Talmy’s state of boundedness

Verb classification is a critical aspect of Chinese grammatical studies (Zhang and Shen, 2017). Shen (1995), from a cognitive perspective, proposed the concepts of “bounded” and “unbounded” verbs, which align with the distinction between dynamic and static verbs in Chinese grammar (Chen, 1988; Gong, 1994; Guo, 1993, 1997). This classification also parallels the English differentiation between durative and non-durative verbs (Quirk et al., 1985; Langacker, 1987, 2009).

Actions occupy a specific duration and can be categorized into “bounded” and “unbounded” types (Shen, 1995 Li, 2011). A bounded action has a defined starting and ending point along the timeline, while an unbounded action lacks these temporal boundaries or may have only a starting point but no clear endpoint (Chen and Xia, 2011). For example, in “I ran to the classroom,” the action starts with “running” and ends at “arriving in the classroom,” making it a bounded action. In “I miss home,” there is no clearly defined starting or ending point for the action, making it unbounded. Bounded actions continue until their endpoint and cannot be arbitrarily extended or shortened. For example, “I ran to the classroom” can be quantified with specific counts, such as “I ran to the classroom once, twice.” In contrast, “I miss home” generally cannot be quantified in this way unless the speaker construes it within a defined temporal boundary, as in expressions like “I often miss home” or “I missed home several times.”

Talmy (2000a) discussed the concept of boundedness within the configurational system, which consists of unboundedness and boundedness. Unboundedness exhibit continuity and indeterminacy without intrinsic finite entity. Boundedness, on the other hand, are discrete units contained within clear boundaries. Boundedness of a verb can often be tested by its compatibility with the grammatical construct “*in* NP_extent-of-time”_:

1. *She dressed in eight minutes.* (The verb *dress* is bounded.)
*She slept in eight hours. (The verb *sleep* is unbounded due to the absence of clear starting and ending points.)

Unbounded verbs, however, can undergo a bounded transformation through specific grammatical structures (e.g., [[_]V _unbd_ + for N _extent of time_]V _bd_). For instance, the example (2) can be transformed into the following.

3. *She slept for eight hours.*
4. *She slept from 3:00 a.m. to 4:00 a.m.*

These modified expressions activate cognitive processes that perceive the action as bounded within a defined temporal framework.

## 3 Data and method

### 3.1 BCC corpus and search results

Talmy (2000b: 27) pointed that a language uses only one of the three types for the verb in its most characteristic expression of Motion, “(1) it is *colloquial* in style, rather than literary, stilted, and so on; (2) it is *frequent* in occurrence in speech, rather than only occasional; (3) it is *pervasive*, rather than limited.” On the other hand, dialogues are prototypical kind of language use in the natural environment, where they can reflect real-time cognitive processing or emotional immediacy. To capture the spontaneous emotional expressions, the materials used in this chapter are all derived from the dialogue sub-database instead of newspapers or literature of the BCC corpus. The BLCU Corpus Center (BCC) is an online corpus primarily focused on Chinese, with additional resources in English and French. It serves as an online big data system for both linguistic ontology research and language application research. The BCC corpus contains approximately 15 billion characters, including materials from various fields such as newspapers (2 billion), literature (3 billion), microblogs (3 billion), science and technology (3 billion), general (1 billion), and classical Chinese (2 billion). It is a large-scale corpus that can comprehensively reflect contemporary societal language use. The BCC corpus boasts several advantages, including its vast data volume, broad domain coverage, and convenient search capabilities. To date, it has supported the publication of hundreds of academic papers.

The research of “often V” and “be often A” (V = verb, A = adjective) in the dialogue sub-database of the BCC corpus were conducted on August 14, 2023, and the results can be found in Table 1.

**Table 1:** Search results for “often V, be often A”.

Retrieval formula	Before filtering	Percentage before filtering	After filtering	Percentage after filtering	Common collocations
often V (经常 V)	40,416	98%	1,508	91%	go, will, eat, come, have, see, suffer from insomnia, do, go on a business trip, say, see, invite, do, stay up late, meet, exchange, get together, drink, cry…
be often A (经常A)	797	2%	147	9%	lonely, bad, painful, aching, head-aching, much, bored, angry, hot, careless, itchy, cold, uncomfortable, busy, excited, sentimental, confused, happy…

The search revealed that “often V” yielded 40,416 results, while “be often + A” yielded only 797, accounting for 98 and 2% of the total results, respectively. This indicates that “often” is most frequently used as an adverb in colloquial contexts to modify verbs, while its combination with adjectives is far less common. After eliminating duplicate data, 1,508 unique instances of “often V” structures and atypical examples were identified, along with 147 unique instances of “be often A” structures and atypical examples. These account for 91 and 9% of the total results, respectively, confirming that “often V” remains predominant even after filtering.

In the “often V” structure, “often” typically functions as an adverb, modifying the verb that follows. This structure is concise and effectively conveys the frequent occurrence of an action or event. It emphasizes the repetition and regularity of actions over a significant period, often appearing in spoken language to describe daily activities, preferences, or experiences. Cognitively, the use of the “often V” structure reflects a temporal understanding, indicating that an action or event occurs frequently in the past, present, and future. This helps listeners form a cognitive model of the speaker’s lifestyle and experiences, aiding in planning activities or predicting behavior.

For instance, expressions such as “I often go,” “I often will,” and “I often do” highlight the speaker’s frequent visits to a place or engagement in an activity, emphasizing the regularity of the events. The key element here is “often” which conveys the speaker’s repeated involvement in certain places or activities and the frequent occurrence of events. This structure is versatile and can be used in various contexts, such as describing personal habits, preferences, or frequently visited places, as well as indicating that something remains common over an extended period.

### 3.2 Mini-corpus and analysis

To further investigate the cognitive semantics and temporal contouring expressions of “customarily X” (X refers to verb or adjective), a mini-corpus with 168 examples was established to examine the dynamic development and usage of “often V” and “be often A.” When establishing the mini-corpus, the raw data were filtered and pre-processed to remove the atypical examples. Four explicit exclusion criteria were adopted, and any case meeting one or more was excluded: (1) if a multi-character word was erroneously segmented into isolated characters, the token was flagged as a segmentation error; (2) if the element following “often” was not a genuine verb or adjective but rather (a) an idiom, or (b) a temporal or quantifier phrase; (3) if the case was bounded by a single event or modified by adverbs such as “suddenly,” hence failing to express a habitual disposition; (4) if the part of speech was incorrect----for instance, verbs mislabeled as adjectives in the raw corpus.

The selection criteria were based on the frequency and typicality. For example, “often go” appeared 3,562 times, ranking the first in the searching, then it would be chosen first in the mini-corpus. The exact example of “often go” will be chosen by discussing with two Chinese teachers who have more than 15 years of teaching experience. The specific search queries and their corresponding frequencies in the mini-corpus are detailed in Table 2:

**Table 2:** Frequency distribution of “often V, be often A” in the mini-corpus.

Retrieval formula	Frequency	Example sentence
often V (经常V)	146 (87%)	I often go.
be often A (经常A)	22 (13%)	Often lonely, so I completely understand.
Total frequency	168 (100%)	

Through the temporal marker “often,” actions, states, and events that occur consecutively are mapped onto the event axis, creating continuity on the timeline. In the “often V” structure, “often” combines with verbs to emphasize the frequency and habitual nature of an action or event, while also expressing emotional experiences over a period of time. This structure is very common in spoken language and has the following characteristics:

- Frequency and habituality of daily actions

The “often V” collocation emphasizes habitual actions in daily life, such as “go,” “will,” “eat,” “come,” “have,” “see,” and other common behavioral habits. These collocations reflect people’s hobbies and behavioral patterns in daily life and are among the most frequent phenomena in everyday life. “often” combined with these action verbs not only indicates repetition in time and space but also represents the evolution of repetitive actions into personal habits or lifestyle choices over time.

b. Expression of emotional experiences

The “often V” structure can also express emotional experiences, such as “often cry.” The verb “cry” indicates that the speaker has encountered something sad and uses crying to express inner feelings, while “often cry” suggests that the speaker has been experiencing sadness recently. Therefore, “often V” reveals the continuity and recurrence of emotional states when used to express feelings.

In the “be often A” structure, “often” is combined with adjectives to describe an individual’s emotional state or physical sensations over a period of time. This collocation is also common in spoken language and has the following characteristics:

- Emphasis on psychological emotions and feelings

The “be often A” can describe a person’s emotional and psychological states over a period, such as “be often lonely,” “be often angry,” or “be often uncomfortable.” These collocations highlight the individual’s emotional experiences and mental states, reflecting the emotions and feelings that people experience after frequently encountering certain events or situations.

b. Description of physical sensations

In addition to emotional experiences, the “be often A” structure is also used to describe physical sensations, such as “be often painful,” “be often itchy,” or “be often cold.” Adjectives like “painful,” “itch,” and “cold” describe the speaker’s physical perception, while “often” emphasizes the speaker’s attention to these bodily states, highlighting their continuity and regularity. This structure conveys the persistence and frequency of physical states over time, reflecting the flow and coherence of time.

To answer Q1, the dialogue sub-database of BCC corpus was used to search the “customarily X” (in forms of “often V, be often A”), and then, the mini-corpus including 168 example sentences is established to study the collocations of the two structures of “customarily X.” The verbs of “often V” are researched in the perspective of cognitive linguistics. To address Q2, we input the collocations into excel and classify the adjectives into several groups, and then try to find the emotions behind these adjectives and examples.

## 4 Results

### 4.1 Cognitive changes of verbs

In the corpus, common collocations with “often V” include verbs such as go, will, eat, come, have, say, look, see, do, stay up late, see, drink, send, buy, listen, go out, be, hit, suffer from insomnia, run, want, and resemble. From a cognitive perspective, these verbs can be categorized into bounded and unbounded verbs.

As an adverb of frequency, “often” collocates with bounded verbs to indicate the repetition of actions. On the time axis, it also signifies temporal repetition, suggesting that the action or event occupies more time. For example, in the conversation:

Speaker 1: “Going to that bookstore again, huh?”

Speaker 2: “Yeah, I often go there.”

Speaker 1: “Next time I’ll go and check it out.”

In the sentence “I often go there,” the action refers to “I often go to that bookstore,” which is omitted by speaker 2 because it is part of the known information mentioned above. The action “I often go” implies both the starting and ending points of the action, which is the bookstore implied in the sentence. Thus, this action is considered bounded. The verb “go” contains both the starting and ending points on the timeline.

**Figure 1:**
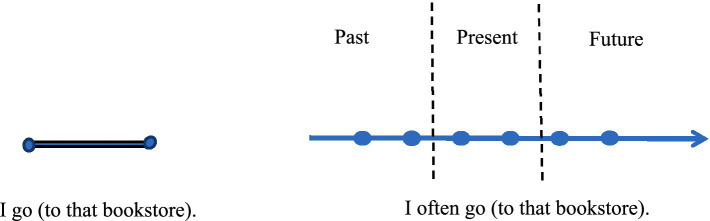
“often + bounded verbs” on the timeline.

In Figure 1, The core schema of “I go (to that bookstore)” is centered on the verb “go” which is a motion event. In contrast, “I often go (to that bookstore)” is characterized by the temporal frequency adverb “often.” Bounded verbs, especially momentary verbs like “go” and “look,” indicate actions that occur briefly and have a short duration. By adding “often,” the experience of time evolves from the past to the present and into the future along the timeline. There are time intervals between the appearance of motion events, which are psychologically perceived as continuations because they recur and happen over a long period of time. The frequency adverb “often” reflects the temporal continuity brought about by habitual actions.

The “often + unbounded verb” changes the state of the unbounded verb, activating the cognitive processing of boundedness. The unbounded verb is metaphorically viewed as an action with a temporal boundary on the timeline, and when paired with specific vocabulary, it becomes a frequent action on the timeline. For example, in the following dialogue:

Speaker 1: “Changed into new clothes? Seeing your outfit reminds me of my school days.”

Speaker 2: “I often have new clothes.”

**Figure 2:**
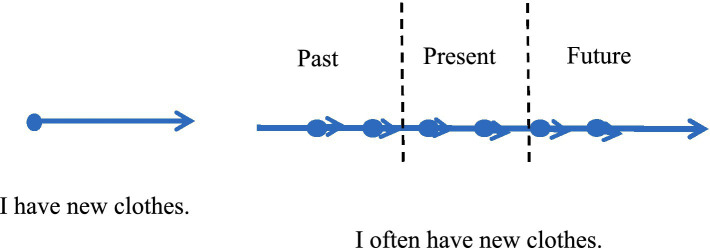
“often+ unbounded verbs” on the timeline.

In this example, “I often have new clothes” is a colloquial expression. The verb “have” contains the start point of the action (when one begins to have new clothes) but lacks an endpoint. The event “have new clothes” may continue into the future, thus “have” is considered an unbounded verb (Figure 2).

Talmy argues that motion events include both displacement events and static events that represent location and existence (Li, 2019). The core representation of “I have new clothes” lies in the unbounded verb “have,” which is a static motion event indicating existence. The verb “have” on the timeline can extend into the future, and any segment of time is “having new clothes,” making it incompatible with adverbs that indicate specific frequencies like “once” or “twice.” However, when the unbounded verb “have” is paired with the time frequency adverb “often,” “have” is divided into actions with temporal nodes in the past, present, and future.

### 4.2 Emotions behind the collocations of “be often A”

After excluding atypical and repetitive examples, a total of 22 frequently occurring “be often A” collocations in dialogues, each appearing 6 times or more, were identified. These adjectives were categorized according to their semantic meanings, resulting in the following list of adjectives and their frequency distribution:

Emotional adjectives: bored, excited, angry, uncomfortable, emotional, confused, happy
Physical sensation adjectives: aching, sore, painful, head-achy, hot, itchy
Personality adjectives: lonely, careless, composed, shy
Quantitative adjectives: insufficient, many
Cognitive and awareness adjectives: unintentional
Evaluative adjectives: bad
Actionality adjectives: busy

**Figure 3:**
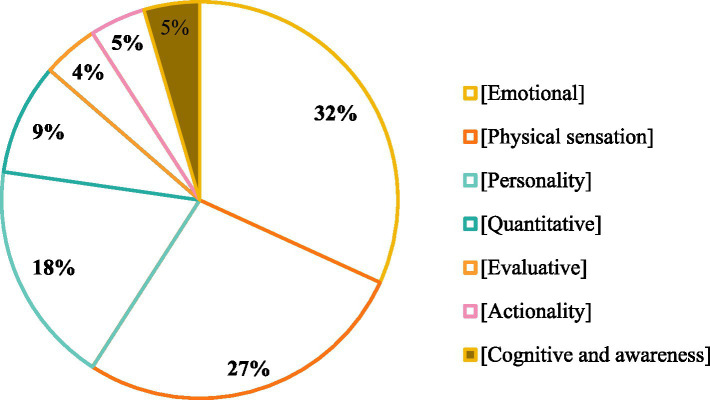
Semantic classification proportions of adjectives.

Figure 3 shows that in the dialogue sub-database, adjectives related to emotional states (32%) and physical sensations (27%) are the most frequent, followed by adjectives related to personality traits (18%). Adjectives related to quantity (9%), cognitive and awareness states (5%), actionality (5%), and evaluations (4%) occupy smaller proportions.

According to the semantic characteristics of adjectives of nature, in terms of what people want, adjectives of nature can also be divided into positive adjectives and negative adjectives (Qi, 2005). In the corpus, most of the adjective collocations of “often A” are negative words, and there are few positive words. According to the emotional feeling of words, the three types and proportion of adjectives in the “be often A” are as follows:

Negative adjectives: bored, angry, uncomfortable, confused, aching, sore, painful, head-achy, hot, itchy, lonely, careless, shy, insufficient, bad.
Positive adjectives: excited, happy, composed.
Neutral words: emotional, many, busy, unintentional.

**Figure 4:**
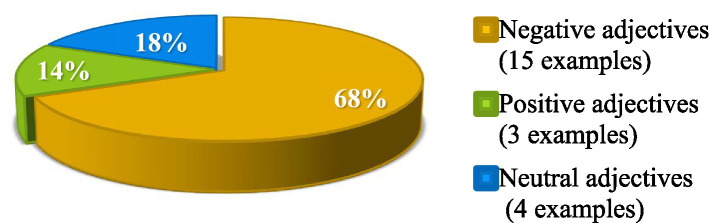
Classification of emotional connotations of adjectives.

From Figure 4, it can be seen that the frequency of the three types of adjectives is not evenly distributed, with negative adjectives (68%) > neutral adjectives (18%) > positive adjectives (14%).

To examine the distribution characteristics of adjective emotional connotations, a Chi-square test was conducted in SPSS 26. The results in Table 3 showed that the Chi-square value was 12.091, with 2 degrees of freedom (df = 2). The asymptotic significance (Asymp. Sig.) was 0.002, which is less than the conventional significance level of 0.05. This indicates that the frequency distribution of the three types of adjectives is not evenly distributed; there is a statistically significant difference.

**Table 3:** Chi-square test results for adjective classification.

Classification	Observed N	Expected N	Residual	Chi-Square	df	Asymp. Sig.
Negative	15	7.3	7.7	12.091^a^	2	0.002***
Positive	3	7.3	−4.3			
Neutral	4	7.3	−3.3			
Total	22					

0 cells (0.0%) have expected frequencies less than 5. The minimum expected cell frequency is 7.3. ***p < 0.01, **p < 0.05, *p < 0.1.

Adjectives expressing physical sensations are all related to unpleasant feelings. For example:

5. [“Do not you eat on time usually?,” “I do, I’m on time at work, but my stomach is often painful.,” “How come?,” “It’s been like this for years, nothing has changed.,” “Poor kid.”]
6. [“Toothache again?,” “I finally decided to have my wisdom teeth extracted.,” “If they hurt constantly, just have them extracted or it will be often tooth-achy in the future.,” “I cannot believe I have four wisdom teeth, and it has not made me any smarter.”]
7. [“I still cannot sleep!,” “How about taking some medicine and drinking some hot water? I am often painful in head and cannot sleep at night.”]
8. [“Have you caught a cold?,” “I am often head-achy since last year.,” “Is it because the wind blows so often?”]
9. [“Is it because of ear mites that your ears are often itchy so much but you cannot scratch them?,” “Yes, it’s been much better since I used the medication.”]

In human cognition, physical pain and discomfort are extremely agonizing and unbearable. People often feel that time moves slowly when experiencing pain, and there is even a sense that time drags on like years. In such moments, people tend to use their “internal psychological time” to gauge “external time.” The examples above (examples 5–9) express bodily pain. In these cases, the speaker’s internal clock runs faster than the physical clock, resulting in a perception of time stretching out. People feel that the time they experienced at that moment was extremely long. Pain serves as an effective regulator of time. When the adverb “often” is used with these pain-related adjectives, it reflects the longer and more frequent psychological imprint of the event in people’s minds. Similarly, adjectives expressing negative emotions, such as:

10. [“How did you find my micro blog?,” “I am often bored and search for other people’s micro blog.”]
11. [“Just stop getting so angry. Look at you, you probably have your face all puffed up from frowning every day.,” “I am not often angry.”]
12. [“Stay strong.,” “I feel. awful.,” “I am also often uncomfortable”].
13. [“I am often confused about who you are.”]

In these examples (10)–(13), the adjectives “bored,” “angry,” “uncomfortable,” and “confused” all express negative emotions. When in a state of sadness or boredom, people find it particularly difficult to get through the time, almost like sitting on pins and needles. These kinds of adjectives, much like those used to describe physical discomfort, tend to leave a lasting psychological impression.

Although positive adjectives are also used to some extent, the sentences in the dialogue express negative emotions or situations that contradict the facts. For example:

14. [“I’ll never believe in love again.,” “Why?,” “Because you have become so serious.,” “I am often composed, okay? Yesterday people were all saying that.”]
15. [“Why are you so excited again? It’s not good to be excited all the time.,” “Am I often excited all the time?,” “Yeah, you are. Calm down, take it easy. You’re a straight talker, aren’t you?”]
16. [“Thanks. Have fun too.,” “I wish you were often happy.”]

In example (14), the word “composed” carries the emotional background of a breakup, which is associated with pain. Here, “composed” refers to being quiet and withdrawn due to a bad mood. In example (15), “excited” indicates an strong emotion, which is generally a positive one like cheer or joy. However, “it’s not good to be excited all the time” points out that excessive excitement can be a problem, which indicates the situation of being over-excited. In example (16), the statement “I wish you were often happy” expresses the speaker’s wish and expectation, even though the person they are talking to is not typically happy, using “often + positive adjective” to express a wish that is the opposite of the truth.

Although 68% of adjectives co-occurring with “be often A” are negative, the adjective polarity alone may not fully capture emotional nuance. Contextual factors such as, co-text or speaker intent are not recoverable from the corpus. Therefore, the percentages reported here indicate lexical tendencies rather than psychological reality.

Our findings align with those of Song, 2021, who, based on her analysis of the Peking University Corpus, observed that “often” tends to co-occur with negative constructions to express a negative state. This may suggest that the frequent pairing of “often” with negative adjectives is not incidental but reflects broader usage patterns in contemporary Chinese. Given the observational nature of this corpus study, follow-up research could combine targeted speaker interviews or rating experiments with pragmatic annotation of conversational contexts to empirically test whether the lexical biases identified here correspond to actual emotional stances.

## 5 Discussion

The primary aim of this study was to explore how expressions related to customarily doing—such as those formed with the frequency adverb “often” —reflect human cognition and emotion. Through an analysis of the Chinese BCC corpus, we observed that such expressions offer significant insight into both habitual actions and emotional states, revealing the underlying cognitive processes that govern language use. One of the most striking findings of this study is that expressions involving “often” are strongly tied to the cognitive representation of time, particularly the conceptualization of habitual actions. The use of “often” in conjunction with verbs of action (e.g., “go,” “eat,” “look”) demonstrates a pattern where habitual behaviors are cognitively processed as repetitive events that span time. These events are conceptualized as recurring intervals along a timeline, reinforcing the idea that habitual actions involve continuous repetition. The interaction between time and habitual action is further underscored in the way language expresses cognitive time. The “customarily X” construction not only reflects habitual actions but also captures the perception of psychological time, wherein time is seen as fluid, cyclical, and even elastic depending on the nature of the experience. When habitual actions or emotional states are expressed, they are often perceived through a lens of continuity rather than discrete moments. The use of “often” thus transforms the meaning of verb and adjective from a singular, momentary action into a temporally extended experience. This cognitive shift indicates that habitual actions are not isolated events, but rather part of a larger framework that reflects how people mentally organize their behaviors and emotional states over time.

Shi and Bai, 2006 pointed out that Chinese views the varying degrees of attributes as a dynamic temporal process, which is why Chinese adjectives share many syntactic features with verbs. In contrast, English treats adjectives as static attributes, lacking the temporal dimension, so English has to utilize verbs to express the dynamic process. In Shi and Bai’s paper, they used the comparative examples to illustrate these differences: English adjectives require a copula (The building *is* high vs. *The building high), whereas Chinese adjectives function directly as predicates (那栋楼非常高 vs. *那栋楼*是*非常高). Therefore, English obligatorily retains the copula (He is often happy), whereas Chinese grammaticalises the adjective as a habitual predicate (他经常开心) in BCC corpus. This difference in conceptualization largely determines the syntactic behavior of adjectives in both languages. In language, emotion is one of the key ways to express an individual’s internal state. People often share their feelings and experiences through words. Emotions can be classified as positive or negative, and the structure “be often A” is more frequently used with adjectives expressing negative emotions. This phenomenon is closely related to how people perceive and express emotions. While the passage of physical time is the same for everyone, negative adjectives tend to occupy a larger cognitive space, as feelings like sadness, pain, and suffering are more enduring and make time feel more difficult to bear. Therefore, when people use the “be often A” structure to describe negative emotions, they are emphasizing the persistence and intensity of these emotions. For example, “be often painful” may not only indicate frequent physical pain but also convey a deep experience of suffering. Similarly, when someone says “be often lonely,” they are not just expressing the experience of feeling lonely often but also sharing this emotion with others, making it easier for others to understand their emotional world.

Overall, the “be often A” structure reflects both the choice of emotion and the concept of psychological time, showcasing how temporal cognition is expressed in language. This structure enriches our understanding of the relationship between time, emotion, and cognition, demonstrating the complexity of language as a tool for thought. Through the “be often A” structure, people can more precisely convey emotional experiences and perceptions of time, with “be often A” better reflecting the psychological time in human cognition.

## 6 Conclusion

Cognition and emotion are closely related to the language we speak. This study provides an in-depth discussion of temporal contouring event with the structure “often V” and “be often A,” examining their semantic and syntactic features, as well as their roles and functions in Chinese. By analyzing different types of “customarily X” (X refers to verb or adjective) structures, a clearer understanding of habitual expressions and phenomena in spoken modern Chinese is achieved.

Firstly, through the study of the semantic characteristics of the word “often,” it is found that it is commonly used to describe the frequency of actions, habitual behaviors, and the normalization of events. Therefore, “often” is not just an adverb of temporal frequency but also reflects human cognitive emotions and sensations. Through the customarily temporal contouring event, people can effectively express habitual events and states.

Secondly, by analyzing specific types of the “customarily X” structure, it is found that in the “often V” structure, the adverb “often” is often combined with directional verbs and action verbs to express the frequent occurrence of an action and its continuation along the temporal axis. This structure is commonly used in modern Chinese to describe individual behavioral habits and repetitive actions. The unbounded verbs in that structure change the state of unboundedness and activates bounded cognitive processing. On the other hand, the “be often A” structure typically describes the frequency of an individual’s emotional state, bodily sensations, or the state of things, and is often associated with negative vocabulary, reflecting the difference between psychological time and physical time.

The research has several limitations that may be acknowledged. First, the corpus-based, observational design limits our findings to correlation (e.g., negative adjectives co-occur with “be often A” most) and not causality (e.g., whether frequent emotional states drive language use or vice versa). Second, although the BCC dialogue sub-corpus is extensive, it is restricted to mainland-Chinese conversational data, much of which originates from online sources. It therefore lacks broader samples of diversified speech. Future research may address these limitations by integrating controlled experiments, such as ERP or eye-tracking, and by expanding the corpus to investigate the cognitive processing of “often V/A.”

## Data Availability

The original contributions presented in the study are included in the article/Supplementary material, further inquiries can be directed to the corresponding author.
