# Supplementary material for: Do the expressions about customarily doing reflect our cognition and emotion: evidence from Chinese BCC corpus
Source: Front Psychol. 2025 Sep 12;16:1545253. doi: 10.3389/fpsyg.2025.1545253 (PMC12463935; doi:10.3389/fpsyg.2025.1545253)
Supplement: Supplementary file 1 [file Data_Sheet_1.docx]

**The micro-corpus of “customarily X”**

**“经常X“的微型语料库**

The corpus is sourced from the BCC dialogue corpus, with a total of 168 example sentences.

语料来自BCC对话语料库，共168个例句。

Search term: often V; There are 146 results in total (the number of occurrences ≥ 40).

# 搜索：经常V；共 146 个结果 （出现的次数≥ 40）

Retrieval time: August 14, 2023

Statistical methods: Random sampling, frequency, and frequency rate Retrieval method: BCC corpus

检索时间：2023.8.14

统计方法：随机取样，频数，频率

检索方法：BCC语料库

Exclude atypical example sentences：

排除非典型例句：

1. ["我这个手机**经常死机**让我对安卓失去了信心",
2. ["你看我像不像会做饭的？","你应该会吧","过年**经常在家**做饭，基本出师了。","棒棒哒，看来我直觉对了"],

Typical example sentences-

典型例句-：

1. ["又去那个书店了啊","嗯那我**经常去** ","下回我也去看看"]
2. "五月份的时候最热了，简直要热疯掉","那七八月呢？","我去年暑假不是没回去嘛，待在这里好像不是很热哦，还**经常会**下雨，感觉还蛮凉快的","这样啊。那为什么五月是最热的？"
3. "要不要见个面啊请你吃饭你在哪","你肯定**经常吃** ","吃饭只是一个形式嘛"
4. ["喜欢的地方就愿意**经常来** ","就是就是"]
5. ["换新装了？看见你装扮我想起了学生时代","新衣服**经常有** ","你常去买衣服啊，消费不少哟"]
6. ["你识我？！！","我认识你啊，但是我不知道你叫什么名字，以前**经常见** 你","在哪见？","学校啊"]
7. ["我好久没有做梦了","经常做梦...","**经常失眠**","为什么会失眠...我最多最多两点就不睡不行了","我经常两点过睡然后五六点就醒了好可怕"],["万一和别人有约的话岂不是更惨了！","只能推掉了…………","对啊真要推掉的话会觉得好对不起人家",
8. ["你这是樱桃还是车厘子？","不都是一种东西"],["真是偷懒的孩纸.","**经常干**这事，以后的机会不多了"],
9. ["今天算了下也想转联通，不过算了笔账有点尴尬，移动刚推出年包是300元一年每个月5G，本来打算转B站22卡，貌似5G也够我用，不过移动话费贵，22卡用一个月也差不多50，移动也算上年包平摊还是每个月50","**经常出差**，国内流量比省内贵很多，联通经常送国内流量的","对，要是出差还是这个好。不过我省内然后对比下资费差不多就犹豫换不换了"],
10. ["例如我向别人借钱借27还30。别人借我钱27只还给我25","**经常遇到**这样的"],
11. ["再下三四场雪差不多","等你哦回来吃肉哦"],["我不忙什么时候给你说我忙啦","不忙喝酒走"],["多谢撒","可以和你成为好朋友不?","**经常交流**哈"],
12. "最近，没和凉粉们出去走动?","**经常聚**啊！"],["你想拉个垫背的是么","必须的！","满满的算计，你俩说好的感情呢","这就是感情啊！死也要一起死"],
13. "才开始装，是找的熟人装修公司，我爸什么事都听他们的，选家居样式都是听他们的，我气死，钱也花了，一点都不好看，不满意，我爸还**经常请** 他们吃饭"
14. ["你们**经常说**方言还是普通话？？？","普通话描述不出来的用方言"]
15. ["你也爱看小说么","是啊！**经常看**。"],["我困了","那就早点睡吧，不过我现在睡不着"],
16. ["有目标性的做点公益活动……","是个办法"],["阴天怎么破","阴天。。。我们这边还下雨了，早上下到现在都没停过，月亮的影都不一定能看见"],["是的，交作业时说忘带了，然后跑出去藏在台阶上赶作业","那我告诉你，我还**经常看到**他早上在这个台阶上吃小面，你是不是也有相同经历？","酷帅的童年是不会当街吃小面的，要吃就吃辣条"],
17. ["你的身体扭变形了","哪里变形，这个动作我**经常做**","感觉脖子要扭断了","并不会而且你说的这个不是我，那是老板娘。"]
18. ["还不睡觉啊？","打算做代购。所以在做跟代购有关的工作。哈哈","代购这样的工作就是要**经常熬夜**吗？","我看别人是。我下月去！所以多做点功课","嗯嗯，多做功课是对的！"],
19. ["今天太高兴啦，以后我们要**经常见**","嗯呐肯定的"],["喊我吃饭马上就出来","好哪天有空出来吃饭"],
20. ["这个点为什么还不睡觉","在外面喝酒…","你最近是**经常喝**酒嘛，看来酒量提高不少。","回家么，唱歌不喝酒总感觉没意思。现在可以喝六瓶了。","厉害利害","…这就厉害了，我们同事都是两件"],
21. ,["搞不清楚，想看却找不到，原来看你**经常发**一些资源，所以想问问有没有。","好像是不好找了，我刚找到一个没法下载。明天回单位给你找找","非常感谢"],
22. ["我下午有看到！不知有没有同一本！你**经常买**书么？","还好，只对感兴趣的会买","我也是偶尔买！看到想看的会忍不住想看完！你只看这种的吗？","不是，我什么都爱看","有什么好看的可以介绍下的呢？","我喜欢八卦"],
23. ["喜子早","早安！"],["卡农，钢琴版的也蛮不错，以前**经常听**","钢琴曲也喜欢"],
24. ["为啥没有圈互娱","互娱和小米直播圈了都！在最后而已！","哈哈"],["下雨哎，人不多吧","不多，差不多都是摄友","现在**经常出去**拍照吗？","没有呢，很少，没有伴","自己去呗"],["别慌，马老师最棒！","还是慌慌的，看一些家长的表情很严肃，不知道是不是对我很不满意"],
25. ["啊？！顿时我没有安全感了～传说，坏蛋干了坏事都喜欢来偏远的地方，例如说，新疆……","哈哈，你是不是想多了","我危机感比较重……不过最让人担心的就是你了，出去一定要结伴～","放心啦！以前我**经常是**晚上一个人走路的，现在不会了","嗯嗯！这就好多了～","你也要注意安全！"],
26. ["小时候我**经常打**我弟","你个坏人你好意思说。","哪个弟弟没有一个打他疼他的姐姐呢打才是爱！","我可是只有疼他。只有被他打哭的份。"],
27. 你电话没改吧！","没改啊，回来提前说啊，我最近**经常跑**厦门"],
28. ["我平时上班都**经常要**走两万步。反而放假却走的少了。","………什么工作啊走这么多","土建工程师。就是管施工单位的工作。","噢噢噢体能有点好"],
29. 反正上次我到了都快三点了你可以定一个机场附近的宾馆，出机场打车去宾馆，或者直接在机场别出来凑合一夜我那天去了之后凌晨在街头游荡了好久","对呀我算了一下不如坐高铁划算所以不准备买这个了"],["哈哈，我**经常玩**手机玩到坐公车坐过站","我也是……哈哈"],
30. ["锦州烧烤好吃，可以多吃点","满大街烧烤店，来嘛？"],["嘿！好久不见","没有好久，我**经常换**头像。","哈哈哈我发现了头像更新的还听频繁"],
31. ["天气冷了也骑着去上班啊","嗯，是的，这边的交通很不方便的..然后我们又**经常加班**到很晚..还是有个车子方便啊..你开始上课了吧？","快乐，休息的有点久了，你晚上回去注意安全呀","嗯嗯...会的，嘻嘻"],
32. ["开车去学校，我晕。好牛","嘿嘿，我爸的车","我**经常坐**11号车，即环保又不堵车，想停就停想走就走，也不怕扣分不怕给罚单"],["听说，看见你了","啊？什么时候？","吃东西的时候","哦哦，也不叫我"],
33. ,["祝你早日拖单","来拯救我","怎么拯救，给你表白啊，让你喝五瓶洗发水"],["我周末**经常回家**","好你回家没有别的事忙可以叫我"],
34. ["我也想吃寿司","走着"],["没有过保的话貌似可以加点钱换个新的","好的，我看看手机啥时候崩","苹果店的人说充电要用自己原装的头，不要用其他的，充电宝也少用","对对我**经常用**充电宝呢"],["你回来啦？","你发毛病时候我不就讲我回来了嘛"],
35. ["请问这种面包要揉出手套膜吗？","应该要的哦"],["我爸**经常带**它出去玩，所以只要我爸一换衣服就很激动，知道马上要出去玩了~","我们家都是我牵出去有时候我妈妈。有两次差点丢了。吓死我了","所以我都不敢松绳子啊！！！"],
36. ["镇雄在全国都有名的，帮派很大的，他们**经常上**云南新闻的。","这种时候你还有心情地域黑也真是厉害的","吃炸洋芋你来对了"],
37. ["祝福祝福","谢谢，话说最近在路上**经常遇到**你","我怎么不知道。。","可能我比较低调，今天就看到你两三次了","难道我很高调吗？真的是，，，","还好吧，就是高跟鞋有点高调","海院不止我一个人穿好吗？"],
38. ["哈哈还是那么喜欢吃番薯干","从来没有改变过，这嗜好"],["换发型了？","就是剪短了呀","是说看着很不一样","是吗上次见你好像也差不多这个长度"],["我说。我**经常看见**你哦。","是嘛。我怎么都没看到你","你没见过我嘛。"],["不知道颜色爱不爱，松紧带略长，可调整…买一赠一","厉害了"],
39. ["世界上还有比你更可爱的人吗","没了"],["这份工作需要**经常出差**吗？看起来很辛苦的样子。","基本都是在出差"],["选择手机验证码登录，输入手机号和验证码，这样比较快","谢谢幻幻，刚刚自己按照提示去做了，可惜没中，只有一元代金券，就不知道下一步了。"
40. ["你喜欢潮一点？还是传统一点成熟一点儿的？","指哪方面呀？、我不懂你的意思哈","各方面吧，例如说穿着之类的","哦，穿着我个人是不喜欢潮的，因为我潮不起来哈。我喜欢运动型的，**经常穿**运动服。","那你是什么专业？","管理类的、","经管？？"],
41. ["摸摸头，每个人都要经历一段这样的时光，想家了，就打电话吧","以前还不怎么想，但是现在会**经常想**家，感觉在这个城市很没有存在感","或许是没有融进校园吧，刚开始都这样，孤独，没有存在感，慢慢就好了，其实大家都一样"],
42. ["你就幸福着吧，可以美美的吃饱饱睡觉觉已经是很大的幸福了。你的幸福**经常出现**在别人的眼里","可以被吵醒也是种幸福亲","行，手机别关机，我醒的时候叫你","好吧，我就挑静音而已"],
43. ["我居然能发图","？？怎么做到的"],["最近没什么好的电视剧看，又重头开始看〈还珠格格〉一二部了，我是不是太无聊了","这事我**经常干**","我也是"],["赞啊，哪里还有这样的学生，给我来一打","嗯，再来一打也可以！"],
44. ["这么说我还挺有远见的。。哈哈，其实就是我个人比较乐观。朋友心情不好也**经常找**我聊天。。所以就萌生了这个想法","嗯嗯。。。那你就好好考虑下咯。。哈哈。。。","看来可以考虑啊。。呵呵~~谢谢师姐啊！",
45. "一堆堆的！而且以前卖的好便宜啊。。三块钱五块钱都有15粒这样！现在一块钱一粒好贵","那真的太贵了。。还记得以前还有阿姨推小车卖寿司的","哈哈哈我也记得，以前你**经常请**我吃东西。。还有美味思奶茶鱼蛋那些",
46. ["真的很喜欢海","所以你**经常能**看到海么","就是不能常见到所以才有种特别的爱","你在内陆城市啊"],
47. ["北京这几天有雷阵雨，局部地区大雨","最近北京**经常下雨**"],["逢考必过！","然爷果然好'这么快回复"],
48. ["哇，我好久没做梦了，睡眠质量太棒","我**经常做梦**"],["为什么不剪空气刘海！","没你帅不敢剪！"],
49. ["那能停车吗，我上次没找到","在往前走一点，修车厂内。"],["最近**经常听到**有人去看这电影。","都是念旧的人被题目给吸引的吧","哈哈是啊。不过我没看过。","蛮值得看的可以看看，","没人和我去电影院看哦","要不要这么惨，找朋友去嘛","不爱出门。","你还是朋友？","我啊。","切那还好意思说！等着网上出了再看吧。"],
50. ["你可以**经常访问**下其他人，比如说我","哦哦哦我试过啊啊啊但是顶不下去","哦不，频率还不够高","怪我了！","不怪你，怪客服"],
51. ["…你也在日照（我是你长岛校友）","我是放假到日照玩，你也是长岛的？"],["新鲜核桃？","嗯这种吃了不苦"],["你**经常睡**这么晚","最近作息有变好啦。。","我没有"],["好友周末好心情，五一节快乐！","五一节快乐！"],
52. ["注意身体","你也要**经常锻炼**，动起来"],
53. ["我也爱我妹妹，家里有两个孩子感觉会热闹很多。虽然小时候我们也**经常吵架**但很快就会和好",
54. 你要**经常回来**看我们啊"
55. "可以阿，我们部门同事**经常拿**东西放到办公桌上，大家一起吃，怎么突然问这个",
56. ["我手机**经常没**信号，移动的。。。。。。"
57. "那不远啊。只是那个家不是**经常回**。周五有时间嘛？跟我打球去","
58. ["哈哈又是梦小盼**经常梦见**以前的同学","哈哈，盼弟，是吗我以前也是老会梦到同学"],
59. "南方那我就不太清楚了……我**经常走**京港澳去北京。有过2次超速警告，也有过超速罚款扣分。
60. "没有了！姐也希望我能有更好的发展是吧？以后还可以**经常见面**的呀","当然希望你更好，加油！"
61. ["啊，我明天回家","哈哈！以后我们**经常联系**吧。说说自己的生活"],
62. "是啊，冬天**经常下**大雪，河北也下雪吧","今年冬天的雪还没下呢",
63. ["头发都掉光了不出家还能干啥","我也**经常掉**头发，不知道为什么"],
64. ["因为我夸的大都是你，所以你觉得我**经常夸**人吧","我没发现，你好像见人都在夸的，"]，
65. ["回来说话，吃大餐","我上半年**经常回去**"],
66. ["手机背面耐挂吗","不拿刀刮，我**经常放**包里，还好","平常不带保护套是没问题的吧","我没带，手感很润滑","打算入手一部最担心就是背面刮花"],
67. "以前坐公交车**经常让**座，
68. "你上学时**经常叫**外卖吃？"
69. ["啊，你设置个每几分钟自动保存，我还是**经常忘记**保存","而且我很郁闷的是，电脑给我了一个修复文件，根本打不开！！
70. ["真羡慕，都在家乡工作，可以**经常可以**出来聚聚，我却孤身在外。"
71. "出息，其实我在学校也是一个人的。**经常觉得**很孤单。寝室人都是三天在学校四天不在我也都是独来独往不过可能比你好点很自由。"],
72. "对啊多出来走动啊","我还是**经常出来**的，没碰到你们，","你几乎不来","我悄悄的来，悄悄的走。。。。。不给你说。",
73. ["我十分想知道，这样的桌布会不会要**经常洗**","需要，所以不常用不过也看你放什么桌子啦","餐桌，一直没勇气",
74. "学校里有好多猫，这只喜欢自己一个猫静静在食堂门口趴着，不爱睁眼，非常酷，所以我**经常拍**它","你可以偷偷抱走他！！","抱了也养不了心塞以后工作了再养猫吧",
75. "冬季家里是不是要**经常开**空调的呢？"
76. "四川这次地震和三峡大坝有没有影响我不知道，我只知道大坝蓄水量起来之后，大坝周围的地方会**经常发生**一些小地震，一般都在四级左右，还没发生过大的地震，我住的地方有震感，是不是和大坝有关我也不知道，只是陈述事实。我家距离三峡大坝三十分钟车程。"],
77. 最近网络**经常断**线都不敢上了"]
78. ["一看这老师也**经常刷**抖音啊","我们数学老师说他不刷抖音的","他当初是不是想当语文老师来着"],
79. 我老公也是**经常出**任务，我压根就不知道他又上哪去干嘛去了，都是说保密，要么就说别瞎打听。
80. ["我也是**经常写**作业，所以我才不会像你一样呢！
81. 只要周末她回这边住，我要是做饭她就会**经常过来**吃饭，跟我说一些很奇奇怪怪的事情。
82. ["我也**经常点**外卖，从来没有给过差评，也不会催单，什么时候送我就什么时候吃，怕晚就早点点外卖，你对人家好，人家自然就对你尊重了"
83. "我们**经常到**那边吃烤鱼"
84. "最重要的是超市**经常搞**活动，搞特价啊，哈哈……"]
85. "小编就是出门**经常忘**带充电器的，不用开机接上通用的数据线到笔记本就可以了。"]
86. ["我哥哥在小时候也欺负我","我小时候**经常欺负**我妹妹。","不带这样的","她乐意"],
87. "我**经常想起**您对我的帮助，心里很温暖。谢谢您！感恩！
88. "我幼年也体质不好，**经常生病**，现在好多了，一半是调理的，一半是运动的",
89. ["而我之前上班骑车只需要五分钟，我还是**经常迟到**","很绝望了"]
90. "妹子的抗压能力不比你们差","做业务需要**经常喝酒**","呃，喝酒至少我不行"],
91. "知道你**经常偷**东西，别说出来"],
92. ["你**经常打电话**吗，效果怎样？","打一百个有一个有意向吧"],
93. ["我最近也**经常戴**帽子，懒得收拾了"
94. "我**经常骂**她啊，但是她甘之如饴"
95. ["我**经常笑**说明我很开心,那你为啥不开心啊","不知道呢，莫名的有时",
96. ["荣耀9屏幕闪屏严重吗？天马屏幕到底如何呀，亮度是不是很低？","亮度高，没有闪屏","他**经常问**别人这句话",
97. ["我也**经常感冒**，吃辣点出身汗就好，多喝点白开水","嗯，这次应该是空调吹到了。"],
98. "恩恩，确实很好吃的说，话说你不是拿它当食堂了么，以后估计**经常碰到**熟人",
99. ["我前年艾特非诚勿扰的一位女嘉宾，后来我们还**经常聊天**呢",
100. 十二点多的火车，据说这趟车还**经常晚点**"
101. "还好啦，我也**经常过去**会展中心"]
102. "说的我都想去翻下留言板看看你们留的言了","我**经常翻**看以前的留言板"
103. 因为我**经常逛**地摊，经常能看见好多男生在那转悠。
104. "我骑着也好惬意哦好开心哦","怀念**经常骑**单车的时光美好"
105. "加油朋友，要**经常运动**"
106. "小姑娘看来**经常跑步**。左腿不细。","这你都能看出来啊！"
107. "以后你会**经常飞**出去...","我想飞进去哦"
108. "喝点醋，我住海边，，小时候**经常卡**住","海鱼不会卡。卡住的都是河鱼"
109. ["终于互动了，希望以后能**经常互动**","哈哈，最近比较忙！都忙晕了头~","忙意味着你被需要，加油"]
110. ["我**经常饿**到晚上回家","一天都不吃嘛。。。","对啊早饭来不及午饭没法吃要上课","摸摸头好可怜"]
111. ["你以前周末不是**经常出门**！！博物馆还逛公园什么的","上周我出了！去了美术馆还看了大世界！","你看！你比爱我更爱美术馆！！"]
112. "那我只能说你是怎么样的人你所理解的世界就是怎样的。我**经常帮**没有零钱的人买巴士票，所以我认为这是非常正常且美好的事。并且在英国，50磅找不开也是常事。"
113. "我**经常丢**东西，公交卡都掉了两张了，还有单位的门进卡都掉了3张了，我都快受不了我自己了"
114. ["两对PVC手套显然不够哇，这绿色核桃染色可厉害了","还有洗手液","估计一时半会洗不掉，小的时候**经常爬**到树上摘核桃吃","嘿嘿，我们的洗手液效果很好","买完了没？我也来一份","谢谢支持，还有"]
115. "以后不在一起上班但是还是要**经常聚**一起吃饭逛街"
116. ["这首歌军训吃饭的时候**经常唱**，感觉算军歌里面好听的了","对的，朗朗上口有气势"]
117. "现在冬天了只要不**经常晒**太阳不久就会恢复"]
118. ["我发现你**经常改名**字","观察真仔细，说，是不是暗恋我？"
119. ["打水的时候一定要注意。水瓶**经常爆**","不是的是在寝室坐着一提就爆了"
120. ["真的吗真的吗疼了好几天了嗓子好像有个东西卡在那里我今天就照你这个吃吃","那应该是发炎了。我嗓子**经常发炎**所以这套方法特别有用，这几天你就把水果当饭吃吧。再不行还是要看下医生啦。"]
121. "我一直用的那个..但是我不**经常化妆**","好吧好吧"
122. "哇哈哈哈……你平时**经常健身**么","没有哇"
123. "你可以自己搜集起来做一个合集，加油，你做好了我就去关注你，请**经常更新**"
124. "啊。。我还以为昨天凌晨呢。他**经常回复**我呀"
125. "对呀!下次一起出来玩","你**经常出没**在解放碑一带嘛"
126. ["哈哈~~你现在还经常踢球吗？我还有**经常打球**哦~~哇哈哈哈~~","过来我这边打吧，球场一直都开着。。。。我都没去打"]
127. "主要是不太习惯，还是比较喜欢中餐","我也是啊","那你**经常煮**中餐吗？"
128. "有兄妹真好！","是好呀，**经常抢**东西哈哈"
129. "我最近脑壳又要不够用了，**经常犯**错误"
130. "银色的不够低调……以前我喜欢银色，现在**经常拍照**觉得黑色方便。"
131. "我最近就**经常哭**，一点小事就很容易生气，然后越想越气，然后一个人默默气哭"
132. "我老公在工作时从来不吃饭，尝菜就尝饱了，老板**经常提醒**他要注意身体。"
133. "太好了。终于不用跟你一起去看电影了。","你**经常陪**他看电影？"
134. "我小时候**经常抓**青蛙玩，我们不觉得没什么。你对青蛙忌讳也可以理解"
135. "那你就种那种食用芦荟吧，万一死了还可以拿剩馀的吃。或者仙人掌，放太阳下面就行，不要**经常浇水**。"
136. ["望才女早日回归健康","已经没事了，谢谢关心","没事就好，我会**经常关注**你的，希望你在创作的同时，也注意一下养生方面的知识"]
137. "我朋友圈有个人**经常卖**二手手机"
138. "没看比赛我平时也**经常耍**到半夜才睡"
139. ["大理会下雪吗","大理的苍山**经常下雪**"]
140. ["现在会**经常吐**吗？","天天吐"]
141. ["头发扎起来我都没认出来","只有在学校里才**经常扎**头发"]
142. ["你是职业旅行家吗？","不是，我是翻译，**经常参加**一些论坛活动，所以跑的地方多些","好厉害的"]
143. ["有时候梦里发生的现实真的会发生这种事情在我身上发生好多次了跟预知一样","我也是，**经常感觉**一些事发生过"]
144. "嗯嗯我的头发也经常掉也比较容易分岔于是就**经常剪** 发尾"
145. "我哥哥一家人**经常喂**小区里的流浪猫。吃得可好啦！"
146. ["哇哈哈哈哈哈哈哈哈哈哈，你会当网红吗？","当然不会","但是你可能要火了呀"],["妹妹叫声好甜呐!!","**经常像**鸽子那样咕咕叫，但是太好动了根本拍不到"]，

Search term: the often A; There are 22 results in total (the number of occurrences ≥ 6).

# 搜索：经常A；共 22 个结果 （出现的次数≥ 6）

Retrieval time: August 14, 2023

Statistical methods: Random sampling, frequency and frequency rate. Retrieval method: BCC corpus

检索时间：2023.8.14

统计方法：随机取样，频数，频率

检索方法：BCC 语料库

Exclusion of atypical example sentences:

排除非典型例句：

1. ["骆世宇**经常长**款","他比我短款还多!!!"]
2. ["昆明也不冷，冬天里**经常大**晴天，欢迎左手姐姐来云南"
3. "某牌手机才买每半年，**经常黑**屏死机，所以准备年底换手机"
4. "时间会证明一切的，我和我闺蜜**经常好**久不联系，但是心里都有彼此，好朋友并不是要天天在一起，硬挤进去的圈子只会自取其辱"],
5. ["因为有钱，**经常美**容，所以很好","说得也是"],
6. "我们**经常小**打小闹～"
7. ["今年过年也没看见你啊","已经走啦"],["我都**经常紧**啦...为毛我就不能回家捏"
8. "你管我，姐姐最近脑袋**经常短**路"
9. "看来适当要运动下……坐姿要正，是不是平时看电视或者在床上**经常歪**着坐的"
10. "谁叫你要**经常鬼**叫啊？"
11. ["**经常慢** 跑不容易饿","昨晚刚快走6.5公里，没用吗"]
12. ["异地一年，从最开始的每天互道晚安但现在的**经常冷** 战莫不关心，有点坚持不下去了","加油"],
13. "饮水机要**经常清**洗的，要换滤芯的，既麻烦，也花钱不少"
14. "高二了，好累啊，我也**经常突然**很伤感……","赞同赞同！！什么事都不如高一那么轻松，学习如此，处事也罢","是啊，高一跟高二对此太轻松了，但高三更累……"]

Typical example sentences-

典型例句-：

1. ["好久没见你啦，争取这个月能面基","么么哒感觉暂时还不太方便出来等我能出门见人的时候联系你"],["孤独是很难受的忍耐，疯了吗还享受","你就不懂了吧，苦中作乐。","**经常孤独**所以太能理解了","一个人的时候其实可以流出很多时间做自己喜欢做的事。"],["大家都去哪里玩？","我可以说我坐在家里看别人玩吗"],
2. ["哎呀~第二个为紫芸报名哒~赞！！！","你不认识我了","**经常粗线** 的小编一般都会眼熟，你是改名换头像了吗也可能是小编脑子不大好使了"],
3. 我再也不相信爱情了","为什么","因为你都会深沉了","**经常深沉**好不好，昨天都还有人在说哈哈哈","那是他们不懂你","你懂我","不懂","就是哦"],
4. ["我现在都不敢乘电梯了我们公司的升降电梯也**经常坏**","有这么夸张么！旁边有红色按钮，掉了就按","升降电梯按啥"],
5. "你平时没按时吃饭吗？","有的，在公司很准时，胃还是**经常痛**","怎么会这样","好几年了，一直未变过","可怜的孩子"],
6. ["牙又疼了？","我终于下定决心把智齿拔掉了~","如果一直疼就拔了吧不然以后会**经常疼**","我居然有四颗智齿，也没见我多聪明"],
7. ["==我到现在都睡不着！！","==吃点药喝点热水怎么样？我也是**经常头痛**晚上睡不着。
8. "我觉得苹果的耐用一点，我的用到现在除了是2g网速这点差评还有内存****经常不够****，其他都挺好的"
9. ["感冒了？","我从去年就开始**经常头疼**","是不风经常吹的"],
10. ["你怎么找到我微薄的","我**经常无聊** 去搜索人家微薄"]
11. ["少生气就行了哇。看你的样子，多半是天天气鼓鼓的把脸胀起来的。","我没有**经常生气**哇"]
12. 我的脸皮可是相当薄啊，**经常害羞**的
13. ["你..........是不是有一万年没说过话了?","明明经常出没的我！比你**经常多** 了！"]
14. ["**经常热**的睡不着","你内火太旺哈哈哈哈"]
15. ["我就是经常遇到这样一个人，不认识，但是**经常不经意**的遇到。我们现在都相视而笑了。"
16. ["是不是因为耳螨所以耳朵**经常痒**想挠又挠不到啊","嗯，这几天用药好多了"
17. ["坚强","我觉得我……难受","我也**经常难受**但是起码现在不难受的时候就不难受平平淡淡"],
18. "你在学校**经常忙** 吗"
19. ["你咋又激动了？经常激动可不好饿","我**经常激动**么？","嗯嗯，经常。放慢心态，平和一点，你该是个有话直说的人吧"
20. "因为看你**经常感慨**","我是个多愁善感的女孩子呀哈哈哈"
21. "我**经常困惑**你是谁？
22. ["thanks～桃子也要玩得开心～","你要是**经常开心** 就好了"]

注：此处形容词分类借鉴了赵春利（2006）《形名组合的静态与动态研究》一文。

“生气”在《现代汉语词典（第7版）》（2016：1168-1169）中的词性和词义为：（动词）因不合心意而不愉快；（名词）生命力、活力。赵春利（2006：162，205） 在替换“骄傲”的形容词和129例情感形容词与名词组合规律时都包含了“生气”一词，且在此文中提到，Aarts 和Calbert（1979）认为大多数形名组合有一种谓语结构，比如“生气的人”。因此本章把“生气”一词归入了“经常A”进行分析。

“感慨”在《现代汉语词典（第7版）》（2016：424）中的词性和词义为：（动词）有所感触而不禁慨叹。赵春利（2006：205）在129例情感形容词与名词组合规律时包含了“感慨”一词。武玉芳（2003：11）认为“感慨”属于变量形容词。在BCC语料里，“感慨”归属为“经常A”类，因此本章在筛选“经常A”时保留了“感慨”一词。

“粗线”，据百度百科记载，该词为网络用语，“出现”的谐音，引申含义有“神经粗大条，不拘小节”，“粗线条”的缩略形式。在《现代汉语词典（第7版）》（2016：221）中，“粗线条”有形容词词性，表示粗略。

“不够”在《现代汉语词典（第7版）》（2016：107）中的词性和词义为：（动词）在数量或条件上比所要求的差些；（副词）表示程度上比所要求的差些。在国学·汉语词典（https://www.hgcha.com/cidian/feb0c9b3.html）和汉语国学·汉语词典（https://www.hanyuguoxue.com/cidian/ci-1cd6e35ee7）中将“不够”划分为形容词词性。因此，本章在筛选时保留了“不够”一词。
